# Telitacicept for autoimmune nephropathy

**DOI:** 10.3389/fimmu.2023.1169084

**Published:** 2023-06-05

**Authors:** Jingjing Cai, Dan Gao, Dongwei Liu, Zhangsuo Liu

**Affiliations:** ^1^ Department of Integrated Traditional and Western Nephrology, the First Affiliated Hospital of Zhengzhou University, Zhengzhou, China; ^2^ Research Institute of Nephrology, Zhengzhou University, Zhengzhou, China; ^3^ Henan Province Research Center for Kidney Disease, Zhengzhou, China; ^4^ Key Laboratory of Precision Diagnosis and Treatment for Chronic Kidney Disease in Henan Province, Zhengzhou, China

**Keywords:** B-cell activating factor BAFF, a proliferation-inducing ligand APRIL, telitacicept, lupus nephritis, IgA nephropathy

## Abstract

B cells and the humoral immunity are important players in the pathogenesis of autoimmune diseases. BAFF (also known as BLYS) and a proliferation-inducing ligand APRIL are required for the maintenance of the B-cell pool and humoral immunity. BAFF and APRIL can promote B-cell differentiation, maturation, and plasma cell antibody secretion. BAFF/APRIL overexpression has been identified in several autoimmune diseases such as rheumatoid arthritis, systemic lupus erythematosus, IgA nephropathy, etc. Telitacicept, a novel fully human TACI-Fc fusion protein that binds both BAFF and APRIL, was approved in China in March 2021 for the treatment of systemic lupus erythematosus at a recommended dose of 160 mg/w subcutaneously and is in clinical trials for the treatment of multiple indications in other autoimmune diseases. In this review, we explored telitacicept’s mechanism of action and clinical data. In addition, the immune features of autoimmune nephropathy were discussed, emphasizing lupus nephritis, IgA nephropathy, and membranous nephropathy.

## 1 Introduction

According to a national cross-sectional survey conducted in 2018-2019, the prevalence of chronic kidney disease in China is 8.2%, affecting 82 million people, which is down from 10.8% a decade ago, but the epidemiological situation remains grim (1, 2). Although the proportion of metabolically related secondary kidney diseases such as diabetes and hypertension is increasing (3), the proportion of immune-related kidney diseases such as lupus nephritis, IgA nephropathy, and membranous nephropathy remains high (4). Autoimmune abnormalities play a vital role in the development and progression of autoimmune nephropathies, and immunomodulation is an important strategy for the treatment of these diseases. In the past, treating autoimmune nephropathy mainly relied on hormones and immunosuppressants. However, with the in-depth exploration of the pathogenesis of autoimmune nephropathy and the rapid development of biomedical research, monoclonal antibodies, such as rituximab and belimumab, have been increasingly applied to kidney disease. As a new biological agent, telitacicept has preliminarily shown good therapeutic effect in the clinical studies in the fields of systemic lupus erythematosus, IgA nephropathy, myasthenia gravis, rheumatoid arthritis, Sjogren’s syndrome, etc. This review summarized and analyzed the pharmacological mechanism, metabolic characteristics, and clinical application of telitacicept. It also comprehensively presented the application of telitacicept in autoimmune nephropathy and its future application prospects.

## 2 Autoimmune nephropathy

**Figure 1:**
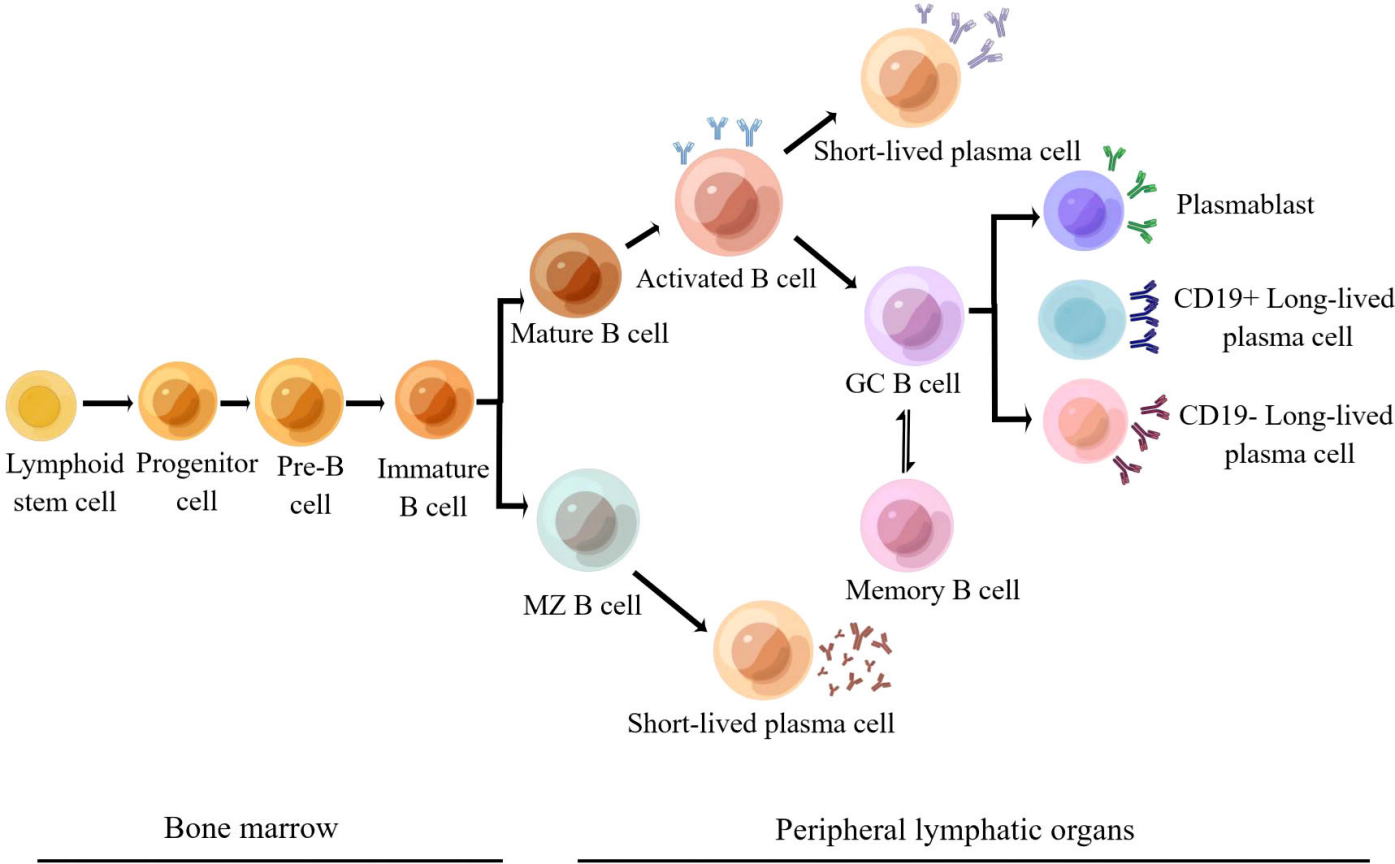
Development and differentiation of B2 cell lines (6) (Adapted from Schrezenmeier et al. By Figdraw).

Many renal diseases have been linked to autoimmune damage, such as lupus nephritis, IgA nephropathy, autoimmune membranous nephropathy, anti-neutrophil cytoplasmic antibody-associated glomerulonephritis, anti-glomerular basal-membrane glomerulonephritis, and C3 nephropathy. B cells play a critical role in the initiation and progression of autoimmune nephropathy. They can differentiate into plasma cells and secrete autoantibodies that act specifically or non-specifically on kidney antigens to form immune complexes and then cause kidney damage (5). B cells undergo several developmental stages in the bone marrow, such as progenitor B cells, pre-B cells, immature B cells, and mature B cells (**Figure 1**). Mature B cells, also known as initial B cells, reach the B-cell region of peripheral immune organs and settle down, where they receive the stimulation of foreign antigens, activate, proliferate, and further differentiate and mature into plasma cells and memory B cells (7). Plasma cells include short-lived and long-lived plasma cells. Short-lived plasma cells secrete a large number of autoantibodies, which contribute to the outbreak of autoimmune diseases. Long-lived plasma cells (also known as autoreactive plasma cells) reside in the bone marrow and inflammatory tissues. They can continuously secrete autoantibodies to maintain the chronic inflammatory process without relying on antigenic stimulation or the assistance of B and T cells. Long-lived plasma cells are resistant to traditional immunosuppressive agents and biologics that target CD20 B cells and are associated with difficulties in treating autoimmune diseases (8). B cells can be divided into B1 and B2 cell lines according to whether they play innate or adaptive immune functions. B1 cells belong to innate immune cells, while B2 cells are the primary cells that secrete antibodies and participate in the humoral immune response (7). A number of cytokines are involved in the development and differentiation of B cells, of which BAFF and APRIL are key factors. Both BAFF and APRIL are members of the tumor necrosis factor ligand superfamily. BAFF and APRIL have two receptors: TACI (transmembrane activator and calmodulin cyclin ligand interaction factor) and BCMA (B-cell maturation antigen). In addition, BAFF can bind to a third receptor, BAFF-R (also called BR3). These receptors are usually expressed by immune cells of the B cell lineage (9). BAFF is a transitional and mature B-cell survival factor that explicitly binds B lymphocytes, co-stimulates their proliferation, and promotes the survival of splenic B cells *in vitro* (10, 11). APRIL mediates IgA class transformation and recombination and maintains the survival of plasma cells (including long-lived plasma cells) (12, 13). High expression of BAFF and APRIL in patients with autoimmune diseases favors the survival of plasma cells, which leads to sustained and enhanced production of autoantibodies that can result in kidney and other tissue damage (14). The pathogenesis of lupus nephritis, IgA nephropathy, membranous nephropathy, and BAFF and APRIL’s role in developing the disease are discussed below.

### 2.1 Lupus nephritis

Systemic lupus erythematosus (SLE) is an autoimmune disease that can affect multiple systems throughout the body, with approximately 60% of cases involving the kidneys, known as lupus nephritis (LN) (15). The pathogenesis of lupus nephritis is multifaceted and incompletely defined, and it is currently believed that sex hormones and environmental exposures can lead to immune system dysfunction in genetically susceptible individuals, such as overreaction of B and T cells, loss of immune tolerance to autoantigens, deficiencies in antibody production and clearance, circulation and tissue deposition of immune complexes, activation of complement and cytokines, and kidney damage (16). Michelle and DC et al. found that serum BAFF levels were higher in SLE patients than in healthy controls and that BAFF levels were positively correlated with CD19+ B cell percentage and the MEX-SLEDAI disease activity score (the Mexican version of the lupus disease activity classification standard) in SLE patients. Serum APRIL levels were also higher in SLE patients than in healthy controls and positively correlated with MEX-SLEDAI and SLICC (International Clinical Collaboration classification criteria) scores (17, 18). The above results are consistent with previous findings of elevated BAFF and APRIL levels in lupus-susceptible mice with SLE (19–23). Matthias et al. discovered that APRIL and BAFF mRNA levels were significantly increased (12-fold and 30-fold, respectively) in the glomeruli of patients with proliferative lupus nephritis, as were tubule interstitial expressions of APRIL, BAFF, BCMA, and TACI (24). Bertrand et al. showed that, compared with control mice, mice treated with the selective APRIL inhibitory antibody Apophe had significantly reduced proteinuria, glomerular cell reduction, and PAS-positive substance deposition at the age of 6 months. There was no significant difference in morbidity and mortality between Apophe-treated mice and control mice 8 weeks after the treatment was stopped (25). Furthermore, studies showed reduced mortality and lower serum IgM, IgG, and anti-DNA antibody levels in APRIL-deficient lupus nephritis mice. Therefore, targeting BAFF/APRIL is expected to be a new strategy for treating lupus nephritis (26). BAFF’s monoclonal antibody, belimumab, specifically neutralizes BAFF. Several large, randomized, controlled phase III clinical trials compared the safety and efficacy of belimumab to standard treatment protocols, demonstrating significantly superior efficacy while maintaining a similar safety profile. This suggests that using targeted BAFF to treat lupus nephritis is a good idea (27–29).

### 2.2 IgA nephropathy

The most common primary glomerular disease worldwide is IgA nephropathy (IgAN), which is characterized by mesangial proliferation and IgA deposition in the glomeruli (30). Although the pathogenesis of IgAN has not been fully elucidated, the theory of multiple attacks is widely accepted. Genetic predisposition factors and abnormal intestinal mucosal immunity lead to increased IgA1 (Gd-IgA1) levels of abnormal glycosylation in individual circulation. In addition, the body produces anti-glycan antibodies that can recognize Gd-IgA1. Abnormally elevated serum Gd-IgA1 and specific anti-glycan antibodies form immune complexes and deposit in renal tissue, leading to proliferation of mesangial cells and extracellular matrix, secretion of cytokines and chemokines, and activation of the local complement bypass pathway, resulting in renal injury (31). Therefore, Gd-IgA1 production is at the core of the pathogenesis. However, the mechanism of Gd-IgA1 production is not fully elucidated. It is believed that innate immune activation mediated by toll-like receptor 9 (TLR9) is involved in the production of Gd-IgA1 (32, 33). McCarthy et al. reported elevated serum and intestinal lamina propria IgA levels in BAFF-overexpressing transgenic mice, and IgA deposition was found in the glomerular mesangium (34). W. Li et al. found that serum BAFF levels were positively correlated with IgA1 levels and mesangial IgA deposition density in IgAN patients (35). Xin et al. revealed that serum BAFF levels were increased in IgAN patients and were associated with clinical and pathologic features of the disease (36). The expression of the APRIL gene in the tonsil germatogenesis center of IgAN patients was increased and correlated with serum Gd-IgA1 level and disease severity. These findings imply that both BAFF and APRIL may be responsible for the creation of Gd-IgA1 (37). Makita et al. looked into the relationship between BAFF/APRIL and TLR9 activation and discovered that APRIL is important in TLR9-induced nephritis-induced IgA overproduction and IgG-IgA IC formation. TLR9 activation increased APRIL gene expression and serum levels. In spleen cells, serum abnormal glycosylated IgA levels were correlated with BAFF and APRIL expression levels, while serum IgG-IgA IC levels were matched with APRIL expression levels but not with BAFF expression levels (38). As a result, targeting BAFF/APRIL could become a new therapeutic strategy for IgAN.

### 2.3 Idiopathic membranous nephropathy

Membranous nephropathy is the most common pathological type of adult nephrotic syndrome, affecting approximately 30% of nephrotic syndrome patients (39, 40). Idiopathic membranous nephropathy (IMN) is responsible for at least 80% of all cases of membranous nephropathy (5). Idiopathic membranous nephropathy is an autoimmune glomerular disease caused by circulating autoantibodies against glomerular podocyte antigens (M-type phospholipase A2 receptor PLA2R and type 1 thrombospondin domain-containing 7A THSD7A). It is characterized by the deposition of large amounts of immune complexes on the epithelial side of glomerular capillary loops. PLA2R antibodies are found in approximately 70% of adult IMN patients, while THSD7A antibodies are found in approximately 2% of adult IMN patients. The sensitivity and specificity of PLA2R antibodies for the diagnosis of IMN are 0.78 and 0.99, respectively. Since antibodies are derived from plasma cells that are differentiated by B cells, B cells play a key role in the pathogenesis of idiopathic membranous nephropathy (41). BAFF and APRIL are involved in the differentiation and survival of B cells and in the conversion of immunoglobulin classes. Their overexpression is involved in various autoimmune diseases, however, the role of BAFF and APRIL in the pathogenesis of IMN and their association with the prognosis of IMN have not been clarified (23, 42, 43). Seung et al. discovered that plasma BAFF levels in IMN patients were higher than in healthy controls, while APRIL levels were comparable. Furthermore, BAFF levels were higher in relapse patients than in the control group, while APRIL levels were higher in non-remission patients. BAFF and APRIL expression levels, like those of other autoimmune diseases, are linked to renal prognosis (44). Rituximab has emerged as a new treatment option for refractory IMN in recent years. Ruggenenti et al. discovered that approximately 30% of IMN patients did not respond significantly to rituximab, which may be due to long-lived memory plasma cells that do not express CD20 (45). Telitacicept inhibits APRIL binding to long-lived plasma cells lacking CD20 expression as well as antibody production. This opens up a new avenue for telitacicept in the treatment of refractory IMN.

## 3 A BAFF/APRIL dual inhibitor - telitacicept

### 3.1 Pharmacology

**Figure 2:**
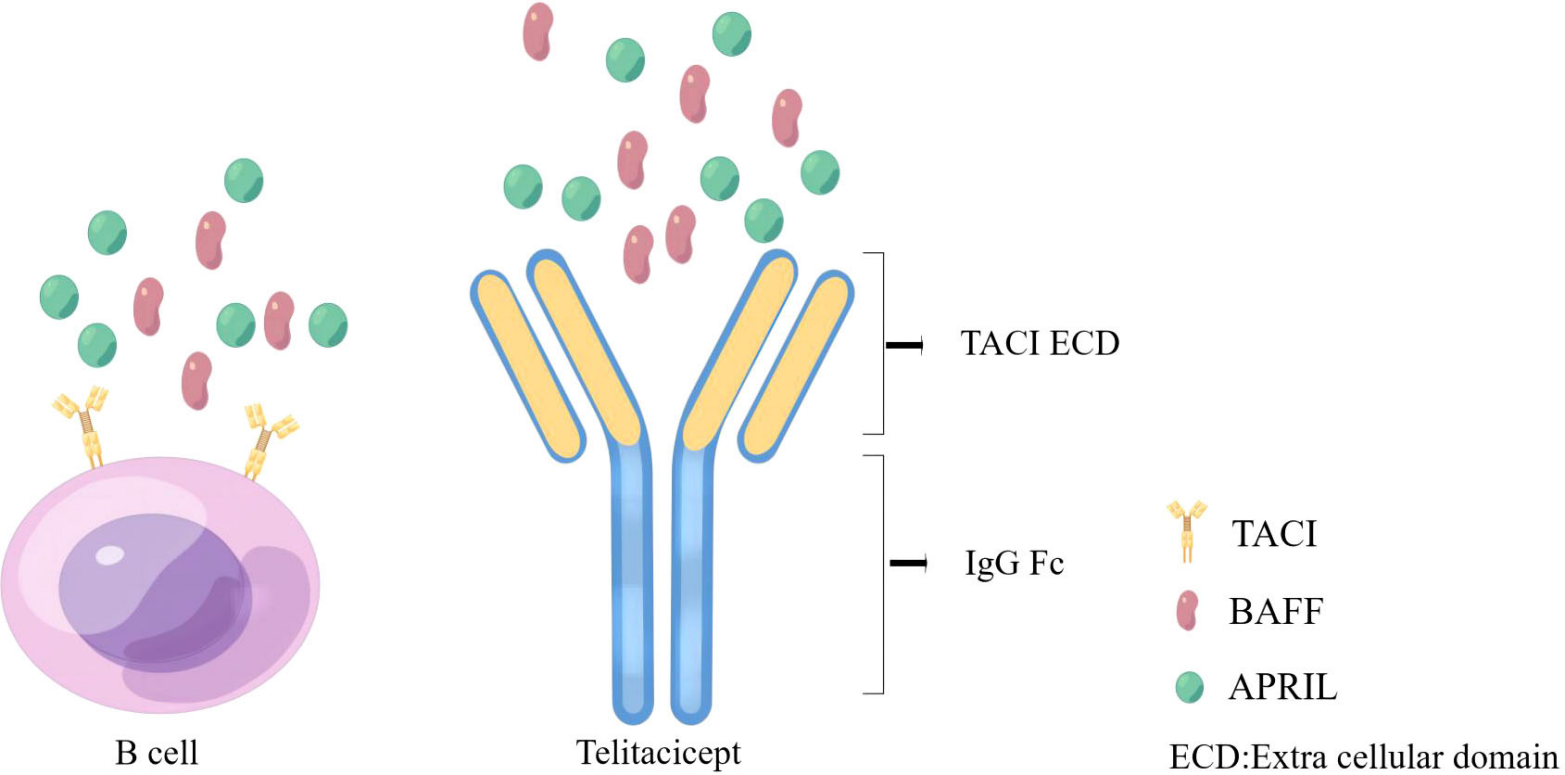
Mechanism of action for telitacicept (By Figdraw).

It is well known that B lymphocytes play a crucial part in the complex pathophysiology of autoimmune nephropathy. Therefore, inhibiting the production of pathogenic antibodies by B cells has become a therapeutic strategy for treating autoimmune nephropathy. BAFF is more involved in the development and maturation of B cells, while APRIL is mainly involved in activating mature B cells and the process by which plasma cells produce antibodies. Anti-BAFF monoclonal antibodies and recombinant fusion proteins (immunoglobulin Fc+TACI) are currently available as targeted therapies against BAFF and APRIL (21, 46). Telitacicept is a new full-human TACI-FC fusion protein prepared by using recombinant DNA technology to connect the extracellular segment of the receptor TACI on the surface of B cells and the Fc segment of IgG1 (**Figure 2**). It can bind BAFF and APRIL, effectively blocking their binding to the receptor (47). The immature B cells can be prevented from continuing to develop and mature by blocking BAFF, which is useful for preventing the recurrence of the condition. Blocking APRIL can prevent mature B cells from differentiating into plasma cells and impact the release of autoantibodies by autoreactive plasma cells, which can effectively manage the symptoms of the disease.

### 3.2 Pharmacokinetics

Currently, studies on the pharmacokinetics of telitacicept have been completed in patients with rheumatoid arthritis (RA), stable systemic lupus erythematosus, and healthy volunteers.

#### 3.2.1 Pharmacokinetic profile of a single ascending dose of telitacicept in Chinese patients with rheumatoid arthritis

Linear

After normalizing the dose of telitacicept by body weight, the serum exposure total telitacicept and free telitacicept (i.e., the area AUClast and plasma peak concentration Cmax under the drug-time curve from time zero to the last quantifiable point) were linearly correlated with the weighted normalized dose of telitacicept. Combined with the elimination half-life, apparent clearance (total telitacicept 9.33~11.58 L/d) and apparent volume of distribution (total telitacicept 199.9~274.9 L) were observed in patients taking 180~540 mg telitacicept. In this dose range, total telitacicept and free telitacicept exhibited linear pharmacokinetics.

Absorption

The total serum telitacicept increased rapidly after single subcutaneous administration, and the median time for reaching the peak was 1 to 2 days in all dose groups. For example, when the dose was 180 mg, the mean serum peak concentration of free telitacicept was 929.9 ng/mL, and the mean peak time was 1.1 days.

Elimination

The mean terminal half-life of total telitacicept increased from 13.3~14.4 d at a low dose to 17.0~32.8 d at 180~540 mg. The BAFF-telitacicept complex elimination half-life increased with increasing telitacicept dose, indicating a shift from targeted clearance in the 1.2-18 mg telitacicept dose group to non-specific clearance in the 60-540 mg telitacicept dose group (48).

#### 3.2.2 Pharmacokinetic characteristics of three different administration regimens in patients with RA

Serum total telitacicept and free telitacicept concentrations peaked within 1~1.5 days after each dose. The plasma peak concentration (Cmax) of the BAFF-telitacicept complex was 28987 IU/mL, 29329 IU/mL, and 65,919 IU/mL, respectively, for the three administration schemes (180 mg BIW, 180 mg QW, and 360 mg QW). The times to peak (Tmax) were 45.5 d, 56 d, and 40 d, respectively. The BAFF-telitacicept complex was cleared by zero-order pharmacokinetics (49).

#### 3.2.3 Pharmacokinetics of multiple doses of telitacicept in patients with SLE

Following multiple doses of telitacicept, total and free telitacicept reached its maximum serum concentration (Cmax) within 1 to 2 days. The mean elimination half-lives of total telitacicept and free telitacicept are 11.4~26.4 and 2.4~26.5 days, respectively (50).

#### 3.2.4 Pharmacokinetic characteristics of telitacicept in healthy Chinese subjects

The median time for total telitacicept concentration to peak was 0.5~1 d in the 80~240 mg dose group, and the peak time was earlier in the low-dose group. The elimination half-lives of the three doses were roughly the same, ranging from 10.9 to 11.9 days. The median time for free telitacicept concentrations to peak was 1 day, and the clearance half-life increased slightly with increasing dose. The BAFF-telitacicept complex had a median peak time of 1557 days, and the peak time increased significantly with increasing dose. In the 160-240 mg dose range, free telitacicept demonstrated linear pharmacokinetics (51).

### 3.3 Comparison with other biological agents

Direct targeting of B cells – targeting CD20 receptors, such as the type I antibody rituximab and the type II antibody obinutuzumab, can largely eliminate peripheral B cells, including memory B cells, but not CD20- pre-B cells and plasma cells (short- and long-lived plasma cells). Indirect targeting of B cells – targeting B cell survival factors such as BAFF, as with belimumab, can directly inhibit B cell maturation and indirectly inhibit plasma cell maturation (short-lived plasma cells), but has no inhibitory effect on long-lived plasma cells. Simultaneous targeting of the B cell survival factors BAFF and APRIL, such as atacicept and telitacicept, can inhibit the transformation of immature B cells into mature B cells and mature B cells into plasma cells and promote the apoptosis of plasma cells (including long-lived plasma cells) (**Table 1**).

**Table 1:** Comparison of biologics directly and indirectly targeting.

	Molecular structure and mechanism of action	Disease in reference	Effectiveness and Safety	Applicable disease
rituximab	It is a human-mouse chimeric monoclonal CD20 type I antibody that specifically binds to the CD20 antigen on the surface of pre-B and mature B lymphocytes and initiates an immune response that mediates B-cell lysis (massive depletion of peripheral B cells, including peripheral blood memory B cells) (6).	proliferative lupus nephritis (52)	In a phase III clinical trial of lupus nephritis patients, the overall renal response rate at 52 weeks was greater in the Rituximab group than in the placebo group, although the difference was not statistically significant. Additionally, there was no change in the patient’s clinical prognosis despite receiving Rituximab treatment for a full year. Adverse reactions include infusion adverse reactions, infection, etc (52).	Off-label for the treatment of SLE, MN, micropathological nephropathy, etc
obinutuzumab	It is a human-derived type II CD20 monoclonal antibody that targets the CD20 antigen expressed on the surface of pre-B lymphocytes and mature B lymphocytes and mediates B cell lysis. Obinutuzumab induces direct cell death with greater activity and affinity for the FcɣRIII receptor protein than rituximab.	proliferative lupus nephritis (53)	When obinutuzumab was added to standard therapy alone, it resulted in a significantly higher rate of complete renal remission at week 52 than standard therapy alone. The most common adverse event was an infection, with an incidence that was similar to that seen in the control group (53).	Off-label for lupus nephritis
belimumab	It is a human lgG1λ monoclonal antibody specific for soluble human BAFF, which inhibits B cell survival (including autoreactive B cells) and B cell differentiation.	active lupus nephritis (29)	The remission rate of the urine protein-creatinine ratio and glomerular filtration rate at 104 weeks was higher in the belimumab group compared to the placebo group. Infection-related deaths occurred at a similar rate in both the belimumab group and the placebo group (29).	For active, autoantibody-positive systemic lupus erythematosus (SLE) patients 5 years of age and older with high disease activity (e.g., positive anti-dsDNA antibody and low complement, SELENA-SLEDAI score ≥ 8) despite conventional therapy
atacicept	TACI-FC fusion protein	active lupus nephritis (54)	The Phase IIb clinical trial of atacicept did not meet its primary endpoint, although there was a trend toward increased the SLE responder index 4 (SRI4) remission rates at week 24 in the 75 mg and 150 mg atacicept groups. Adverse reactions in the atacicept group were no higher than in the placebo group (54).	Clinical trials of Atacicept for RA, SLE, and others are ongoing (55)
telitacicept	TACI-FC fusion protein	SLE, IgA nephropathy, and others	As mentioned above, stage III SLE and stage IIb IgA nephropathy all showed effectiveness, the adverse reactions were within a controllable range, and symptomatic treatment was required.	Based on conventional treatment, there is still high disease activity and autoantibody-positive systemic lupus erythematosus; clinical trials for systemic myasthenia gravis are ongoing (56).

## 4 Application of telitacicept in autoimmune renal disease

### 4.1 Systemic lupus erythematosus

12 patients with mild systemic lupus erythematosus were randomly assigned to receive 180 mg of telitacicept or a placebo subcutaneous injection on days 0, 7, 14, and 21 in the phase I exploratory investigation. Considering the limited sample size, there was no discernible difference between the telitacicept and placebo groups in terms of SLEDAI scores. Telitacicept, however, reduced peripheral blood lymphocyte counts (CD19+ B cells and IgD+ B cells) and serum immunoglobulin levels in SLE patients (48).

In a phase IIb clinical trial, 249 patients with active systemic lupus erythematosus were randomly assigned to receive subcutaneous telitacicept 80 mg, 160 mg, 240 mg, or placebo (1:1:1:1) once weekly for 48 weeks in addition to standard of care. Telitacicept-treated patients had a significantly higher SLE Response Index (SRI4) response rate than placebo patients at week 48 (71.0% in the 80 mg group, 68.3% in the 160 mg group, and 75.8% in the 240 mg group, p<0.0001). Furthermore, the treatment group reduced the SELENA-SLEDAI score by 4 points more than the placebo group (50.0%) (75.8% in the 80 mg group, 77.8% in the 160 mg group, and 79.0% in the 240 mg group, p<0.001). At week 48, the occurrence of adverse events was comparable across all groups (57).

A self-controlled retrospective study assessed telitacicept’s efficacy and safety in the treatment of children with refractory systemic lupus erythematosus (cSLE). After 5 to 26 weeks (80 or 160 mg per week) of telitacicept, the response rate of SRI4 in 15 refractory cSLE patients was 66.7% (10 cases). In 12 patients, the median hormone dose was reduced from 40 mg/d to 17.5 mg/d. 8 renal impaired patients with urine protein >0.5 g at baseline 24 hours before treatment showed a decrease in urine protein 24 hours after treatment. In 8 cases, 2 urine proteins turned negative, and 5 plasma albumin increased to normal. In addition, 3 of the 8 patients with renal impairment improved renal function to varying degrees (eGFR ml/min·1.73m2, from 17.4 to 26.6, 40.7 to 48.2, and 63.2 to 146.0, respectively) (58).

Preliminary data from a domestic phase III confirmatory study of telitacicept for the treatment of SLE are now available. In combination with standard therapy, 335 patients with SLE were randomly assigned to the telitacicept (160 mg) or placebo groups *via* subcutaneous injection once weekly for 52 weeks. The primary endpoint was reached at week 52, with a significantly higher proportion of patients in the telitacicept 160 mg group achieving SRI4 remission compared to the placebo group (82.6% vs. 38.1%, p<0.001) (59). The global multicenter Phase III clinical trial was approved by the European Union and the National Medicines Administration on September 26 and 28, 2022, respectively (60).

### 4.2 IgA nephropathy

Telitacicept’s IgA nephropathy indication was approved by the US Food and Drug Administration (FDA), which exempted the Phase I clinical trial in the US and conducted the Phase II clinical trial directly. The first patient was enrolled and administered in November 2021. On November 18, 2022, the FDA approved a Phase III clinical trial of Telitacicept in the United States for the IgA nephropathy indication (61). The efficacy and safety of telitacicept in treating IgA nephropathy were initially evaluated in phase II domestic clinical trials. The data showed that after 24 weeks of treatment, urine protein levels were significantly reduced in subjects in the 240 mg group compared to baseline. In addition, the average 24-hour urine protein level was reduced by 49% compared to baseline, which was statistically significant compared to the placebo group (p<0.05). Therefore, it reduces proteinuria in high-risk IgA nephropathy patients and may effectively reduce the risk of progression of IgA nephropathy (62).

### 4.3 Ongoing clinical trials

Seven autoimmune disease indications (including SLE, neuromyelitis optica spectrum disease, rheumatoid arthritis, IgA nephropathy, Sjogren’s syndrome, multiple sclerosis, and myasthenia gravis) are in commercialization or clinical trials (63).

## 5 Telitacicept tolerability

A total of 89 adverse events, mostly mild or moderate, were reported in 12 patients in a phase I trial of multiple subcutaneous injections of telitacicept (180 mg QW*4) in SLE patients, with 14 likely to be related to telitacicept. Musculoskeletal and connective tissue diseases and infections were the most frequent side events, which were significantly higher in the telitacicept group than in the placebo group (7[77.78%] vs. 1[33.33%]) and slightly higher in the telitacicept group than in the placebo group (7[77.78%] vs. 2[66.67%]). The majority of infections occurred within two weeks of the last dose, when the patient’s immunoglobulin level had reached a trough, or after the addition of other immunosuppressive agents to treat SLE exacerbations. One patient in the telitacicept group experienced severe adverse events and reactions, including systemic lupus erythematosus activity index elevation and cholecystitis. Endotoxin shock occurred in one patient. Miliary tuberculosis and traumatic arthritis occurred in one patient. Epiglottitis occurred in one patient. However, none of them resulted in the experiment being terminated prematurely (50). In a phase IIb trial of multiple subcutaneous injections of telitacicept (80 mg, 160 mg, and 240 mg QW*48) in SLE patients, the most common adverse events were upper respiratory tract infections and injection-site adverse reactions. The telitacicept and placebo groups had comparable rates of adverse and severe adverse events (p > 0.05). The study drug was not thought to be responsible for one death in the telitacicept 240 mg group (57). In the domestic phase III confirmatory SLE study, the incidence of adverse events and those leading to trial termination in the telitacicept group was similar to that in the placebo group (153[91.6%]) vs. 142 [84.5%]. 8 [4.8%]) vs. 9 [5.4%]). However, the rate of serious adverse events was lower in the telitacicept group than in the placebo group (12 [7.2%] vs.24 [14.3%]). The most common adverse events were upper respiratory tract infection, decreased blood IgG and IgM, injection site adverse reactions, and urinary tract infections (59).

In a Phase I trial of a single subcutaneous injection of telitacicept in healthy volunteers, 36 subjects were randomly assigned to the telitacicept 80, 160, and 240 mg groups. A total of 42 mild or moderate adverse events were reported by 36 subjects. Elevated blood triglyceride levels, positive urinary white blood cells, and upper respiratory tract infections were all common side effects. Subcutaneous administration was well tolerated, with no unexpected side effects observed (51).

A total of 140 adverse events, all mild or moderate, were reported in a Phase I trial of a single subcutaneous injection of telitacicept (1.2, 6, 18, 60, 180, 360, and 540 mg) in 28 patients with RA. The most common adverse event was upper respiratory tract infection, and the telitacicept group was significantly higher than the placebo group (15 [71.43%]) vs. 3 [42.86%]) (48). In a trial of multiple subcutaneous injections of telitacicept (180 mg QW* 3,180 mg BIW* 8,360 mg QW*5) in RA patients, a total of 146 adverse events, all mild or moderate, were reported in 21 patients. The most frequent adverse effects were injection site reactions and upper respiratory tract infections. The telitacicept group outperformed the placebo group by a large margin (11 [78.6%] vs. 2 [28.6%]). 10 [71.43%]) vs.0 [0%]) (49).

In general, the adverse reactions of telitacicept mainly included infection, musculoskeletal and connective tissue diseases, injection site adverse reactions, etc. Yet, no significant adverse events occurred before the experiment came to an end, and effective symptom management was possible. In addition, a good safety profile was also shown in Phase II trials of telitacicept in IgA nephropathy, primary Sjogren’s Syndrome, and myasthenia gravis (62, 64, 65).

## 6 Conclusion and prospect

The traditional treatment of autoimmune nephropathy mainly includes corticosteroids, cyclophosphamide, mycophenolate mofetil, and other immunosuppressive agents, which can non-specifically inhibit B cells and short-lived plasma cells. However, the efficacy has significant limitations, such as a low complete response rate, a long treatment cycle, and a significantly increased risk of infection and osteoporosis. Therefore, there is an urgent need to develop targeted drugs with better efficacy and safety. Telitacicept inhibits the B-cell survival factors BAFF and APRIL, preserving autoimmunity while exerting therapeutic effects. BAFF/APRIL overexpression is a common characteristic of several autoimmune nephropathies and other autoimmune diseases. So far, telitacicept is effective in clinical trials of SLE, Neuromyelitis optical spectrum disease, rheumatoid arthritis, IgA nephropathy, primary Sjogren’s syndrome, relapsing-remitting multiple sclerosis, and systemic myasthenia gravis (56, 58, 63, 66). It is theoretically possible to demonstrate efficacy in idiopathic membranous nephropathy with abnormal BAFF/APRIL expression in autoimmune nephropathy. However, additional basic and clinical trials are required to confirm this. In addition, searching for appropriate specific treatment individuals (such as abnormal expression of BAFF/APRIL) is also a problem that must be solved before the current application of telitacicept so that patients can benefit more.

## Author contributions

JC and DG contributed to the process of literature review. JC drafted the manuscript. DL and ZL critically revised the manuscript. All authors contributed to the article and approved the submitted version.
